# Proton Pump Inhibitor and Clopidogrel Use After Percutaneous Coronary Intervention and Risk of Major Cardiovascular Events

**DOI:** 10.1007/s10557-021-07219-6

**Published:** 2021-07-09

**Authors:** John Maret-Ouda, Giola Santoni, Shaohua Xie, Annika Rosengren, Jesper Lagergren

**Affiliations:** 1 Upper Gastrointestinal Surgery, Department of Molecular Medicine and Surgery, Karolinska Institutet, Retzius Street 13a, 4th Floor, 17177 Stockholm, Sweden; 2 Centre for Clinical Research Sörmland, Uppsala University, Eskilstuna, Sweden; 3 Department of Molecular and Clinical Medicine, Institute of Medicine, Sahlgrenska Academy, University of Gothenburg, Sahlgrenska University Hospital, Gothenburg, Sweden; 4 School of Cancer and Pharmaceutical Sciences, King’s College London, London, UK

**Keywords:** Clopidogrel, PCI, Percutaneous coronary intervention, MI, Myocardial infarction, GERD, GORD, Gastroesophageal reflux disease, Gastro-esophageal reflux disease, Proton pump inhibitor, PPI

## Abstract

**Purpose:**

Due to shared hepatic metabolism, concomitant medication with a proton pump inhibitor (PPI) and clopidogrel might reduce the effectiveness of clopidogrel in the prevention of cardiovascular events after percutaneous coronary intervention (PCI). We aimed to examine the risk of major cardiovascular events after PCI comparing patients who used clopidogrel together with PPI with those who used clopidogrel alone.

**Methods:**

This Swedish nationwide cohort study included patients who received clopidogrel after primary PCI in 2005–2019. Patients were followed for up to 12 months after PCI. Data were retrieved from the Swedish Prescribed Drug Registry, Patient Registry, Cancer Registry, and Cause of Death Registry. Multivariable Cox regression provided hazard ratios (HRs) with 95% confidence intervals (CIs) for cardiovascular events comparing PPI users (exposed) with non-users of PPI (non-exposed). The HRs were adjusted for sex, age, comorbidity, calendar period, obesity, diabetes, anti-diabetic medication, tobacco-related diseases, hypertension, and congestive heart failure.

**Results:**

The cohort included 99,836 patients who received clopidogrel after primary PCI. Among these, 35,772 (35.8%) received concomitant PPI. Compared to non-users, PPI users had increased adjusted HRs of all study outcomes, i.e., the main outcome myocardial infarction (HR = 1.23, 95% CI 1.15–1.32) and the secondary outcomes coronary heart disease (HR = 1.28, 95% CI 1.24–1.33), stroke (HR = 1.21, 95% CI 1.05–1.40), and death due to coronary heart disease (HR = 1.52, 95% CI 1.37–1.69). The results were similar in analyses including both primary and secondary PCIs.

**Conclusions:**

In patients who receive clopidogrel after PCI, concomitant use of PPI seems to increase the risk of major cardiovascular events.

**Supplementary Information:**

The online version contains supplementary material available at 10.1007/s10557-021-07219-6.

## Introduction

Myocardial ischemia caused by obstruction of coronary arteries is often treated with percutaneous coronary intervention (PCI) where the obstruction is dilated and one or more stents are inserted to maintain the passage of blood. In addition to usually life-long treatment with aspirin, dual antiplatelet therapy with a P2Y12-antagonist is recommended for 12 months after PCI to counteract thromboembolic events, including occlusion of stents which could otherwise cause renewed ischemia and infarction[1, 2].

Proton pump inhibitors (PPI), one of the most common groups of medications globally, reduce the acidity of the gastric contents and are mainly used in the treatment or prevention of gastro-esophageal reflux disease or peptic ulcer disease. PPI is also recommended for gastric mucosa protection in patients with an elevated risk of duodeno-gastric bleeding who require antiplatelet therapy. Some studies have suggested that PPI treatment reduces the efficacy of clopidogrel [3], which may be due to shared cytochrome P450 enzymes (including CYP2C19) and reduced metabolism of the prodrug clopidogrel to the active metabolite (2-oxo-clopidogrel) of clopidogrel [4]. Such reduction in the efficacy of clopidogrel could potentially increase the risk of thromboembolic events after PCI. Existing data on the topic are conflicting, however, which highlights the need for large and population-based studies [3, 5].

The aim of this study was to help clarify if and to what extent concomitant use of PPI increases the risk of major thromboembolic events among patients who use clopidogrel after PCI in a large and unselected cohort.

## Methods

### Design

This was a population-based and nationwide Swedish cohort study during the period July 1, 2005, to December 31, 2019. The source cohort was an updated version of the Swedish Prescribed Drugs and Health Cohort (SPREDH). SPREDH is a Swedish population-based cohort, created with the aim of investigating how specific medications influence various outcomes [6]. SPREDH includes users of commonly prescribed medications, i.e., almost all adult Swedish residents. The data in SPREDH come from the following national health data registries in Sweden: *Prescribed Drug Registry*, *Patient Registry*, *Cancer Registry*, and *Cause of Death Registry*, which are described below. The complete national coverage of these registries, in combination with the well-maintained system with personal identification numbers to identify all Swedish residents, enabled complete follow-up within all hospital and healthcare systems [7]. The study was approved by the Regional Ethical Review Board in Stockholm (permission numbers 2016/982–31/4 and 2018/271–32).

### Data Sources for the Source Cohort

The source cohort (SPREDH) contains cleaned and merged data from four national registries.

- *The Prescribed Drug Registry* contains data regarding all prescribed and dispensed medications in Swedish pharmacies from 1 July 2005 onwards. The prescriptions are directly transferred from the pharmacies to the registry, thereby providing complete nationwide coverage [8]. The medications are defined using the codes in the Anatomical Therapeutic Chemical Classification System (ATC).
- *The Patient Registry* holds data regarding all diagnoses and procedures at all Swedish hospitals or specialist out-patient clinics since 1987. These are coded according to the International Classification of Diseases version 10 (ICD-10). The Patient Registry also contains information about dates of admissions, hospital codes, and patient demography, including age and sex. The data are used for monetary reimbursements within the healthcare systems, administrative purposes, and research [9]. The Patient Registry has a completeness regarding in-patient main diagnosis of 99% and a positive predictive value for most diagnoses and procedures ranging from 85 to 100% [9, 10].
- *The Cancer Registry* contains data on all malignant tumors diagnosed in Sweden from 1958 onwards. Pathologists and clinicians report cancer cases to the Cancer Registry with overall completeness of 96% [11, 12].
- *The Cause of Death Registry* started in 1952 and records the date of death as well as the primary and contributing causes of death in all Swedish residents (including those who die abroad) [13]. The Cause of Death Registry is 100% complete and the validity of the data is high [7].

### Study Cohort

The cohort specifically used for the present study included all adult patients (≥ 18 years) in SPREDH who underwent a first PCI between July 1, 2005, and December 31, 2019 (ICD 10 codes FNG02 and FNG05) and were treated with clopidogrel (ATC code B01AC04) within 2 weeks of PCI. The ICD codes defining the indications for PCI are presented in Supplementary Table 1. Patients entered the study 2 weeks after the date of their PCI and remained in the study until the first occurrence of any of the outcomes, another PCI, death, or end of follow-up (12 months after PCI).

### Exposure

A person was defined as exposed from baseline if that person used PPI (ATC code A02BC) in the year before study entry. After baseline, PPI was considered as a time-varying exposure where patients who dispensed a prescription of PPI after PCI were crossed over from the unexposed to the exposed group at the date of first PPI dispensation after PCI.

### Outcomes

The main outcome was myocardial infarction (ICD-10 code I21). There were four secondary outcomes: new event of hospitalization for coronary heart disease (ICD-10 code I20-I25), ischemic stroke (ICD-10 code I63), all-cause mortality, and mortality due to coronary heart disease (ICD-10 codes I20-I25 as an underlying cause of death).

### Covariates

The study controlled for the influence of eight covariates (with categorizations in brackets): sex (male or female), age (continuous), calendar period (continuous), obesity-related diseases (obesity diagnosis, diabetes mellitus type 2 due to its close correlation with obesity, or use of anti-diabetic medication; no or yes), tobacco-related diseases (bronchitis, emphysema, or chronic obstructive pulmonary disease; no or yes), hypertension (diagnosis of hypertension, elevated blood pressure, secondary hypertension, hypertensive kidney disease, or renovascular hypertension; no or yes), congestive heart failure (no or yes), and Charlson comorbidity index score (0, 1, 2, or ≥ 3). A calendar period was included to take changes in clinical practice during the study period into account. Comorbidities recorded any time before the first PCI were assessed from the Patient Registry as described in the Appendix (Table 4).

### Statistical Analysis

Multivariable Cox proportional hazards regression models were used to calculate hazard ratios (HRs) with 95% confidence intervals (CIs), comparing PPI use with PPI non-use in relation to the outcomes. The proportionality assumption of the Cox regression models was tested on the basis of Schoenfeld residuals and all models satisfied the assumption. The HRs were adjusted for the eight covariates listed and categorized above. One sub-analysis compared different types of PPI in relation to myocardial infarction (the main outcome). Another sub-analysis included not only the primary PCI, but also any additional PCIs. All analyses were conducted by an experienced biostatistician (GS) according to a detailed and pre-defined study protocol, using the statistical software Stata version 15MP (StataCorp, College Station, TX, USA).

## Results

### Patients

The study cohort included 99,836 patients who underwent the first PCI and received clopidogrel. Among these, 35,772 (35.8%) had concomitant PPI treatment and 64,064 (64.2%) did not receive such treatment. Table 1 presents the characteristics of the study participants, divided by PPI use. The most common indication for PCI was myocardial infarction in both groups (63.2% among PPI users and 65.7% in non-users), and almost all of the remaining patients had PCI because of angina. The male predominance was lower among the PPI users (66.3%) than among the non-users (75.4%), and the median age was slightly higher among PPI users (69 years) compared to non-users (67 years). The PPI group had a higher incidence of obesity- and tobacco-related disorders, hypertension, congestive heart failure, and previous myocardial infarction, and also higher Charlson comorbidity scores, compared to non-users of PPI (Table 1).

**Table 1:** Characteristics of patients using a proton pump inhibitor (PPI) together with clopidogrel, and those only using clopidogrel after primary percutaneous coronary intervention (PCI)

Variable	PPI + clopidogrel after PCI	Clopidogrel only after PCI
Total, N (%)	35,772 (100.0%)	64,064 (100.0%)
Sex, N (%)		
Men	23,729 (66.3%)	48,310 (75.4%)
Women	12,043 (33.7%)	15,754 (24.6%)
Age, median (inter-quartile range (IQR))	69 (61–77)	67 (59–75)
Calendar year, median (IQR)	2010 (2008–2013)	2010 (2008–2012)
Indication for PCI		
Angina, N (%)	13,043 (36.5%)	20,957 (32.7%)
Myocardial infarction, N (%)	22,612 (63.2%)	42,101 (65.7%)
Recurrent myocardial infarction, N (%)	168 (0.5%)	139 (0.2%)
Complications following myocardial infarction, N (%)	10 (0.02%)	11 (0.02%)
Other acute ischemic heart disease, N (%)	87 (0.2%)	125 (0.2%)
Chronic ischemic heart disease, N (%)	2524 (7.1%)	3775 (5.9%)
Cerebral infarction, N (%)	90 (0.3%)	119 (0.2%)
Past medical history prior to PCI		
Obesity-related disease*, N (%)	9056 (25.3%)	13,168 (20.6%)
Tobacco-related disease*, N (%)	3001 (8.4%)	3004 (4.7%)
Hypertension*, N (%)	21,638 (60.5%)	33,312 (52.0%)
Congestive heart failure, N (%)	6591 (18.4%)	8526 (13.3%)
Prior myocardial infarction, N (%)	3460 (9.7%)	4418 (6.9%)
Charlson comorbidity score, N (%)		
None	15,440 (43.2%)	36,728 (57.3%)
1	8557 (23.9%)	13,366 (20.9%)
2	4888 (13.7%)	6625 (10.3%)
≥ 3	6887 (19.3%)	7345 (11.5%)

^*^Obesity-related diseases included obesity diagnosis, diabetes mellitus type 2, or use of anti-diabetic medication; tobacco-related diseases included bronchitis, emphysema, or chronic obstructive pulmonary disease; hypertension included a diagnosis of hypertension, elevated blood pressure, secondary hypertension, hypertensive kidney disease, or renovascular hypertension

### Risk of Myocardial Infarction

Table 2 presents the HRs of cardiovascular events comparing patients on clopidogrel for PCI who also used PPI with non-users of PPI. Myocardial infarction occurred in 4.3% (n = 1522) of the PPI users and in 3.3% of non-users of PPI (n = 2134). The HR of myocardial infarction was 23% higher among PPI users compared to non-users (adjusted HR 1.23, 95% CI 1.15–1.32). The analysis of different types of PPI revealed increased HRs of all studied PPIs, but without any clear differences in strength of association between the types (Table 3).

**Table 2:** Hazard ratios (HR) with 95% confidence intervals (CI) of coronary events comparing patients using both a proton pump inhibitor (PPI) and clopidogrel with those only using clopidogrel after primary percutaneous coronary intervention (PCI)

	Patients	Person-years	Cases	HR (95% CI)*
Myocardial infarction				
Clopidogrel	64,064	55,750	2134 (3.3%)	1.00 (Reference)
PPI + clopidogrel	35,772	30,525	1522 (4.3%)	1.23 (1.15–1.32)
Coronary heart disease				
Clopidogrel	64,064	51,800	9534 (14.9%)	1.00 (reference)
PPI + clopidogrel	34,781	27,417	6011 (17.3%)	1.28 (1.24–1.33)
Stroke				
Clopidogrel	64,064	56,400	439 (0.69%)	1.00 (reference)
PPI + clopidogrel	36,046	31,173	349 (0.97%)	1.21 (1.05–1.40)
All-cause mortality				
Clopidogrel	64,064	56,595	1074 (1.7%)	1.00 (reference)
PPI + clopidogrel	36,110	31,355	1434 (4.0%)	1.71 (1.58–1.86)
Mortality due to coronary heart disease				
Clopidogrel	64,064	56,595	713 (1.1%)	1.00 (reference)
PPI + clopidogrel	36,110	31,355	832 (2.3%)	1.52 (1.37–1.69)

^*^Adjusted for sex, age, calendar year, obesity-related diseases, tobacco-related diseases, hypertension, congestive heart failure, and Charlson comorbidity score

**Table 3:** Hazard ratios (HR) with 95% confidence intervals (CI) of myocardial infarction comparing patients using different types of proton pump inhibitors (PPI) and clopidogrel with those only using clopidogrel after percutaneous coronary intervention (PCI)

	Patients	Person-years	Cases	Crude HR (95% CI)	Adjusted HR* (95% CI)
Clopidogrel	64,064	55,750	2134	1.00 (reference)	1.00 (reference)
Omeprazol + clopidogrel	26,789	22,644	1126	1.37 (1.28–1.48)	1.22 (1.13–1.31)
Pantoprazol + clopidogrel	4138	3623	187	1.42 (1.22–1.64)	1.39 (1.20–1.62)
Esomeprazole + clopidogrel	3540	3070	157	1.39 (1.18–1.64)	1.29 (1.09–1.52)

^*^Adjusted for sex, age, calendar year, obesity-related diseases, tobacco-related diseases, hypertension, congestive heart failure, and Charlson comorbidity score

### Risk of Coronary Heart Disease

A new event of non-fatal coronary heart disease occurred in 17.3% (n = 6011) of the PPI users and in 14.9% of non-users of PPI (n = 9534). The HR of coronary heart disease was 28% higher in the PPI group (adjusted HR 1.28, 95% CI 1.24–1.33).

### Risk of Ischemic Stroke

Among the PPI users, 349 (1.0%) had a stroke during follow-up. The corresponding frequency among non-users was 439 (0.7%). The HR of stroke was 21% higher among users of PPI compared to non-users of PPI (adjusted HR 1.21, 95% CI 1.05–1.40).

### Risk of All-cause and Disease-specific Mortality

Among the concomitant users of PPI, 1434 (4.0%) died of any cause, including 832 (2.3%) who died due to coronary heart disease. Among non-users of PPI, 1074 (1.7%) died during follow-up, including 713 (1.1%) who died from coronary heart disease. Comparing concomitant PPI users with those not using PPI, the HR was 71% higher for all-cause mortality (adjusted HR 1.71, 95% CI 1.58–1.86) and 52% higher for mortality specifically due to coronary heart disease (adjusted HR 1.52, 95% CI 1.37–1.69).

### One or More Percutaneous Coronary Interventions

Sub-analyses of all PCIs, independent of whether they were primary or secondary, revealed similar results as those assessing primary PCI only (Supplementary Table 2 and 3).

## Discussion

This study found increased risks of myocardial infarction, coronary heart disease, stroke, and all-cause and disease-specific mortality among patients receiving PPI treatment together with clopidogrel compared to those only using clopidogrel within 12 months of PCI.

Among methodological strengths of the study are the population-based and nationwide design and the complete follow-up of participants, which provided a large sample size, minimized selection bias and losses to follow-up, and facilitated generalizability. Another advantage was the adjustment for several confounders, including obesity-related diagnoses and tobacco-related diagnoses. Yet, a limitation was that residual confounding by these indirectly measured exposures cannot be excluded, and these factors might still contribute to the associations. Additionally, the influence of some other potentially relevant confounders was not possible to adjust for, e.g., lifestyle factors, dietary exposures, or physical activity; we cannot rule out that PPI is only a proxy for other unmeasured factors leading to a reduced risk of cardiovascular events. Another limitation was the lack of data on adherence to medication with clopidogrel and PPI or whether the medication was discontinued on medical grounds. Adherence to clopidogrel should be high given the serious risks associated with non-use after PCI, while the continuing need for PPI use is less certain because not all patients will receive it as primary gastric protection but may use it for treatment of gastroesophageal reflux disease. PPI is available over-the-counter but only in small packages and at a much higher cost for the patient. Thus, patients requiring long-term treatment with PPI receive this through prescriptions. Any discontinuation of PPI use or missed PPI treatment due to over-the-counter medication should occur at random, and thus not explain the associations found, but rather dilute them.

Whether the use of a PPI together with clopidogrel increases the risk of cardiovascular events after PCI compared to the use of clopidogrel alone is a matter of debate. A recent systematic review and meta-analysis of 23 studies and 156,823 patients found that concomitant use of PPI and clopidogrel increased the risk of myocardial infarction (risk ratio 1.43, 95% CI 1.24–1.66), other major adverse cardiovascular events combined (risk ratio 1.22, 95% CI 1.06–1.39), and cardiovascular death (risk ratio 1.21, 95% CI 0.97–1.50) [3]. However, a sub-analysis of six randomized controlled trials (4476 patients) in that meta-analysis showed no increased risk of major cardiovascular events (risk ratio 0.99, 95% CI 0.76–1.28) [3]. The previously largest original study on the topic, a cohort study of 24,702 patients with myocardial infarction, showed that compared to non-users of PPI, the risk of major cardiovascular events was similarly increased among concomitant users of PPI and clopidogrel (HR 1.29 (95% CI 1.17–1.42)) as in PPI users without clopidogrel (HR 1.29 (95% CI 1.21–1.37)) but that study did not only include patients undergoing PCI [5]. The largest randomized controlled trial on the topic included 3761 patients with any indication for clopidogrel and found no difference between the group receiving clopidogrel and PPI and the group receiving only clopidogrel (HR 0.99 (95% CI 0.68–1.44)) [14], but the statistical power was low and the duration of follow-up was short (median of 106 days) [14]. Another observational study including 706 patients, among which approximately two-thirds received clopidogrel, found that concomitant use of PPI was associated with an increased risk of acute ischemic events, heart failure, or death (HR 2.28 (95% CI 1.24–4.18)) [15]. The present study, focusing on cardiovascular events specifically after PCI, is substantially larger than any previous original study on the topic and adds evidence in favor of an increased risk of major cardiovascular events if patients use PPI together with clopidogrel after PCI.

The proposed mechanism behind the associations found in this study is decreased effectiveness of clopidogrel when PPI is administered. Several in vitro studies have shown that PPI and clopidogrel share the metabolic pathway through the cytochrome P450 enzyme (CYP2C19), an enzyme necessary for the metabolism of clopidogrel to its active metabolite [16]. An in vitro study suggested that different types of PPI may affect specific CYP2C19s differently, with pantoprazole being the least potent inhibitor and lansoprazole the most potent inhibitor [17]. This finding prompted our separate analyses of the three most common types of PPI, but these analyses showed no obvious differences in risk estimates. Additionally, a portion of the population is carriers of an allele leading to reduced function of CYP2C19, which has been estimated to affect approximately 30% of the adult Caucasian and Black populations and 60% of Asian populations, and can lead to reduced effectiveness of clopidogrel [18, 19]. Intermediate or poor metabolizers are expected to be represented in the current cohort as well and might have affected the results.

Both the American College of Cardiology expert consensus and the European Society of Cardiology recommend the use of PPI concomitant to treatment with clopidogrel among patients with a history of upper gastrointestinal bleeding as well as among patients with multiple risk factors for gastrointestinal bleeding [2, 20]. Except for these indications of using PPI, the results of the present study suggest that PPI should be used cautiously among patients treated with clopidogrel after PCI, particularly in patients with less clear indications for PPI treatment.

In conclusion, the findings of this large and population-based cohort study indicate that the large group of patients who use PPI together with clopidogrel following PCI are at an increased risk of serious cardiovascular events compared to patients who use clopidogrel only after this intervention. The findings argue in favor of avoiding less necessary PPI treatment during clopidogrel treatment within 12 months of PCI.

### Electronic supplementary material

Below is the link to the electronic supplementary material.

Supplementary file1 (DOCX 19 KB)

## Data Availability

Data is stored within the Karolinska Institutet.

## Appendix 1

**Table 4:** Charlson co-morbidity index without age

Diagnosis	ICD 8	ICD 9	ICD 10	Score
Peripheral vascular disease	93,00–93,99, 440,00–440,99, 441,00–441,99, 437, 00–437,99, 443,00–443,99	093A, 437D, 440-440X, 441-441X, 443-443X, 557B-557X, V43E	I70.0-I71.9, I73.1, I73.8, I73.9, I77.1, I79.0, I79.2, K55.1, K55.8, K55.9, Z95.8, Z95.9	1
Cerebrovascular disease	377, 02, 377,03, 430,00–438,99	362D, 430-438X	G45.0-G45.9, G46.0-G46.9, H34.0, I60.0-I69.9	1
Dementia	290,00–290,99	290-290x, 294B, 331C	F00.0-F03.9, F05.1, G30.0-G30.9, G31.1	1
Chronic pulmonary disease	490,00–493,99, 506,01, 506,09	416 W, 416X, 490-505X, 506E, 508B, 508 W	I27.8, I27.9, J40.0-J47.9, J60.0-J67.9, J68.4, J70.1, J70.3	1
Connective tissue disease	446,30, 734,00–734,98, 712,10–712,50, 716,00–716,99	446F, 710A-710E, 714A-714C, 714E-714X, 725-725X	M05.0-M06.9, M31.5, M32.0-M34.9, M35.1, M35.3, M36.0	1
Peptic ulcer disease	531,00–534,99	531–534	K25.0-K28.9	1
Diabetes, uncomplicated	250,00–250,03 250,08, 250,09	250, 250A, 250B, 250H, 250X	E10.0, E10.1, E10.6, E10.8, E10.9, E11.0, E11.1, E11.6, E11.8, E11.9,E12.0, E12.1, E12.6, E12.8, E12.9,E13.0, E13.1, E13.6, E13.8, E13.9,E14.0, E14.1, E14.6, E14.8, E14.9	1
Diabetes end-organ damage	250,04–250,07	250D-250F	E10.2–E10.5, E10.7, E11.2–E11.5,E11.7, E12.2–E12.5, E12.7, E13.2–E13.5, E13.7, E14.2–E14.5, E14.7	2
Chronic kidney disease	403,99, 404,99, 582,00–583,99, Y29,01	403-404X, 582-582X, 583-583X, 585, 586, 588-588X, v42a, v45b, v56	I12.0, I13.1, N03.2–N03.7, N05.2–N05.7, N18.0-N19.9, N25.0, Z49.0–Z49.2, Z94.0, Z99.2	2
Hemiplegia	343,00–344,99	334B, 342-342X, 343-343X, 344-344F, 344X	G04.1, G11.4, G80.1, G80.2, G81.0-G83.4, G83.9	2
Leukemia and malignant lymphoma	200,00–208,99	200-208X	C81.0–C85.9, C88.0-C88.9, C90.0–C97.9	2
Solid tumor	140,00–172,99, 174,00–195,99	140-172X, 174-195X	C00.0-C26.9, C30.0–C34.9, C37.0–C41.9, C43.0-C43.9, C45.0–C58.9, C60.0–C76.9	2
Solid tumour and metastatic	196,00–199,99	196-199X	C77.0-C80.9	6
Liver disease mild	070,01–070,09, 570,00–571,99, 573,00	070-070X, 570-570X, 571-571X, 573D-X, V42H	B18.0-B18.9, K70.0–K70.3, K70.9,K71.3–K71.5, K71.7, K73.0-K74.9,K76.0, K76.2–K76.4, K76.8, K76.9, Z94.4	1
Liver disease moderate to severe	456,00, 573,02, 573,03	456A-456C, 572B-572X	I85.0, I85.9, I86.4, I98.2, K70.4, K71.1, K72.1, K72.9, K76.5-K76.7	3
AIDS	N/A	279 K	B20.0–B22.9, B24.0-B24.9	6

## Code Availability

Code is available upon request.
