# Supplementary material for: Proton Pump Inhibitor and Clopidogrel Use After Percutaneous Coronary Intervention and Risk of Major Cardiovascular Events
Source: Cardiovasc Drugs Ther. 2021 Jul 9;36(6):1121–8. doi: 10.1007/s10557-021-07219-6 (PMC9652231; doi:10.1007/s10557-021-07219-6)
Supplement: Supplementary file 1 — Supplementary file1 (DOCX 19 KB) [file 10557_2021_7219_MOESM1_ESM.docx]

**Supplementary Table 1.** Indications for percutaneous coronary intervention (PCI).

| **Diagnosis** | **ICD-10 code** |
| --- | --- |
| Angina | I20 |
| Myocardial infarction | I21 |
| Recurrent myocardial infarction | I22 |
| Complications following myocardial infarction | I23 |
| Other acute ischemic heart disease | I24 |
| Chronic ischemic heart disease | I25 |
| Cerebral infarction | I63 |

**Supplementary Table 2.** Characteristics of patients using a proton pump inhibitor (PPI) together with clopidogrel, and those only using clopidogrel after one or more percutaneous coronary interventions (PCI).

| **Variable** | **PPI + clopidogrel**  **after PCI** | **Clopidogrel**  **after PCI** |
| --- | --- | --- |
| Total, N (%)  Sex, N (%) | 43,565 (100.0%) | 73,622 (100.0%) |
| Men | 29,446 (67.6%) | 56,079 (76.2%) |
| Women | 14,119 (32.4%) | 17,543 (23.8%) |
| Age, median (inter-quartile range [IQR]) | 69 (62-77) | 67 (60-75) |
| Calendar year, median (IQR) | 2010 (2008-2013) | 2010 (2008-2012) |
|  |  |  |
| Number of PCIs |  |  |
| 1 | 38,328 (88.0%) | 67,744 (92.0%) |
| 2 | 4,525 (10.4%) | 5,345 (7.3%) |
| 3+ | 712 (2.3%) | 533 (0.7%) |
|  |  |  |
| Past medical history prior to PCI |  |  |
| Obesity-related diseases, N (%) | 11,940 (27.4%) | 16,227 (20.0%) |
| Tobacco-related diseases, N (%) | 3,774 (8.7%) | 3,602 (4.9%) |
| Hypertension, N (%) | 27,424 (62.9%) | 39,865 (54.2%) |
| Congestive heart failure, N (%) | 8,789 (20.2%) | 10,561 (14.3%) |
|  |  |  |
| Charlson comorbidity score, N (%) |  |  |
| None | 18,005 (41.3%) | 40,972 (55.7%) |
| 1 | 10,457 (24.0%) | 15,732 (21.4%) |
| 2 | 6,013 (13.8%) | 7,787 (10.6%) |
| 3+ | 9,090 (20.9%) | 9,131 (12.4%) |

**Supplementary Table 3.** Hazard ratios (HR) with 95% confidence intervals (CI) of coronary events comparing patients using both a proton pump inhibitor (PPI) and clopidogrel with those only using clopidogrel after one or more percutaneous coronary interventions (PCIs).

|  | **Patients** | **Person-years** | **Cases** | **HR (95% CI)*** |
| --- | --- | --- | --- | --- |
| ***Myocardial infarction*** | | | | |
| Clopidogrel | 73,622 | 63,932 | 2,527 (3.4%) | 1.00 (Reference) |
| PPI + clopidogrel | 43,565 | 37,154 | 2,020 (4.6%) | 1.26 (1.19-1.34) |
|  |  |  |  |  |
| ***Coronary heart disease*** | | | | |
| Clopidogrel | 73,622 | 59,528 | 11,128 (15.1%) | 1.00 (Reference) |
| PPI + clopidogrel | 42,423 | 33,449 | 7,736 (18.2%) | 1.31 (1.27-1.35) |
|  |  |  |  |  |
| ***Stroke*** | | | | |
| Clopidogrel | 73,622 | 64,653 | 511 (0.69%) | 1.00 (Reference) |
| PPI + clopidogrel | 43,875 | 37,946 | 439 (1.0%) | 1.24 (1.08-1.41) |
|  |  |  |  |  |
| ***All-cause mortality*** | | | | |
| Clopidogrel | 73,622 | 64,879 | 833 (1.1%) | 1.00 (Reference) |
| PPI + clopidogrel | 43,946 | 38,172 | 1,722 (3.9%) | 1.65 (1.53-1.78) |
|  |  |  |  |  |
| ***Mortality due to coronary heart disease*** | | | | |
| Clopidogrel | 73,622 | 64,879 | 833 (1.1%) | 1.00 (Reference) |
| PPI + clopidogrel | 43,946 | 38,172 | 1,020 (2.3%) | 1.48 (1.35-1.63) |

* Adjusted for sex, age, calendar year, obesity related diseases, tobacco related diseases, hypertension, congestive heart failure, and Charlson comorbidity score.
